# Diverse small molecules prevent macrophage lysis during pyroptosis

**DOI:** 10.1038/s41419-019-1559-4

**Published:** 2019-04-11

**Authors:** Wendy P. Loomis, Andreas B. den Hartigh, Brad T. Cookson, Susan L. Fink

**Affiliations:** 1 Department of Laboratory Medicine, University of Washington, Seattle, WA USA; 2 Department of Microbiology, University of Washington, Seattle, WA USA

## Abstract

Pyroptosis is a programmed process of proinflammatory cell death mediated by caspase-1-related proteases that cleave the pore-forming protein, gasdermin D, causing cell lysis and release of inflammatory intracellular contents. The amino acid glycine prevents pyroptotic lysis via unknown mechanisms, without affecting caspase-1 activation or pore formation. Pyroptosis plays a critical role in diverse inflammatory diseases, including sepsis. Septic lethality is prevented by glycine treatment, suggesting that glycine-mediated cytoprotection may provide therapeutic benefit. In this study, we systematically examined a panel of small molecules, structurally related to glycine, for their ability to prevent pyroptotic lysis. We found a requirement for the carboxyl group, and limited tolerance for larger amino groups and substitution of the hydrogen R group. Glycine is an agonist for the neuronal glycine receptor, which acts as a ligand-gated chloride channel. The array of cytoprotective small molecules we identified resembles that of known glycine receptor modulators. However, using genetically deficient *Glrb* mutant macrophages, we found that the glycine receptor is not required for pyroptotic cytoprotection. Furthermore, protection against pyroptotic lysis is independent of extracellular chloride conductance, arguing against an effect mediated by ligand-gated chloride channels. Finally, we conducted a small-scale, hypothesis-driven small-molecule screen and identified unexpected ion channel modulators that prevent pyroptotic lysis with increased potency compared to glycine. Together, these findings demonstrate that pyroptotic lysis can be pharmacologically modulated and pave the way toward identification of therapeutic strategies for pathologic conditions associated with pyroptosis.

## Introduction

Pyroptosis is a programmed process of lytic, proinflammatory cell death^1^ involved in a host of disorders including sepsis, stroke, intestinal inflammation, and T-cell depletion during HIV infection^2–5^. Although pyroptosis contributes to pathological inflammation and cell death, it is also an essential protective host response to infection^6^. Pyroptosis is mediated by proteases in the caspase-1 family, which are activated by the innate immune signaling platforms termed inflammasomes. Inflammasomes respond to microbial or damage-associated stimuli via pattern recognition receptors in the NOD-like receptor (NLR) and AIM2-like receptor families^7^. Caspase-1 proteolytically converts the proforms of interleukin 1β (IL-1β) and IL-18 to mature inflammatory cytokines. Caspase-1 also cleaves gasdermin D, releasing the N-terminal pore-forming domain, which inserts into the plasma membrane^8^. Gasdermin D pores mediate osmotic cell swelling, rupture of the plasma membrane, and release of intracellular contents including the enzyme lactate dehydrogenase (LDH)^9,10^. The importance of pyroptotic death in the pathogenesis of disease is highlighted by protection of gasdermin D knockout mice from conditions including septic lethality^11^ and autoinflammatory disease^12,13^.

Glycine is a simple amino acid, which prevents terminal loss of membrane integrity during pyroptosis via unknown mechanisms^14^. Glycine does not inhibit upstream caspase-1 activation, pore formation, IL-1β secretion, or loss of mitochondrial membrane potential, but specifically affects the final lytic event, demonstrating that this process can be independently manipulated^9,15,16^. Glycine also has a well-demonstrated cytoprotective effect on cell death resulting from hypoxia and oxidant injury (reviewed in ref. ^17^). In these models, there is no role for glycine metabolism, ATP preservation, changes in cytosolic calcium, intracellular pH regulation, or cytoskeletal stabilization. The presence of glycine during hypoxic injury prevents loss of viability and allows cells to recover respiratory function and ATP levels upon reoxygenation^18^. Although the mechanism underlying glycine protection against hypoxia and oxidant injury is incompletely understood, multiple lines of evidence point to glycine acting as a ligand at an unidentified cell surface receptor^17^. Glycine administration is highly protective in models of sepsis^19–21^, suggesting that understanding the mechanism of glycine action may provide novel therapeutic targets for inflammasome-mediated pathology. In this study, we describe specific structural requirements for glycine protection against pyroptotic lysis. We additionally identify novel inhibitors of pyroptotic lysis with increased potency compared to glycine.

**Table 1:** A subset of ion channel modulators prevent pyroptotic lysis

	Inhibits pyroptotic lysis	GlyR	GABA_A_
Strychnine	Yes	Antagonist/agonist	
Brucine	Yes	Antagonist/agonist	
Nipecotic acid	No	Antagonist	
Pregnenolone sulfate	Yes	Antagonist	Antagonist
Picrotoxin	No	Antagonist	Antagonist
Ginkgolide B	No	Antagonist	Antagonist
Bicuculline	No		Antagonist
Muscimol	Yes		Agonist
THIP	No		Agonist

## Results

### Structural requirements for glycine cytoprotection

**Fig. 1:**
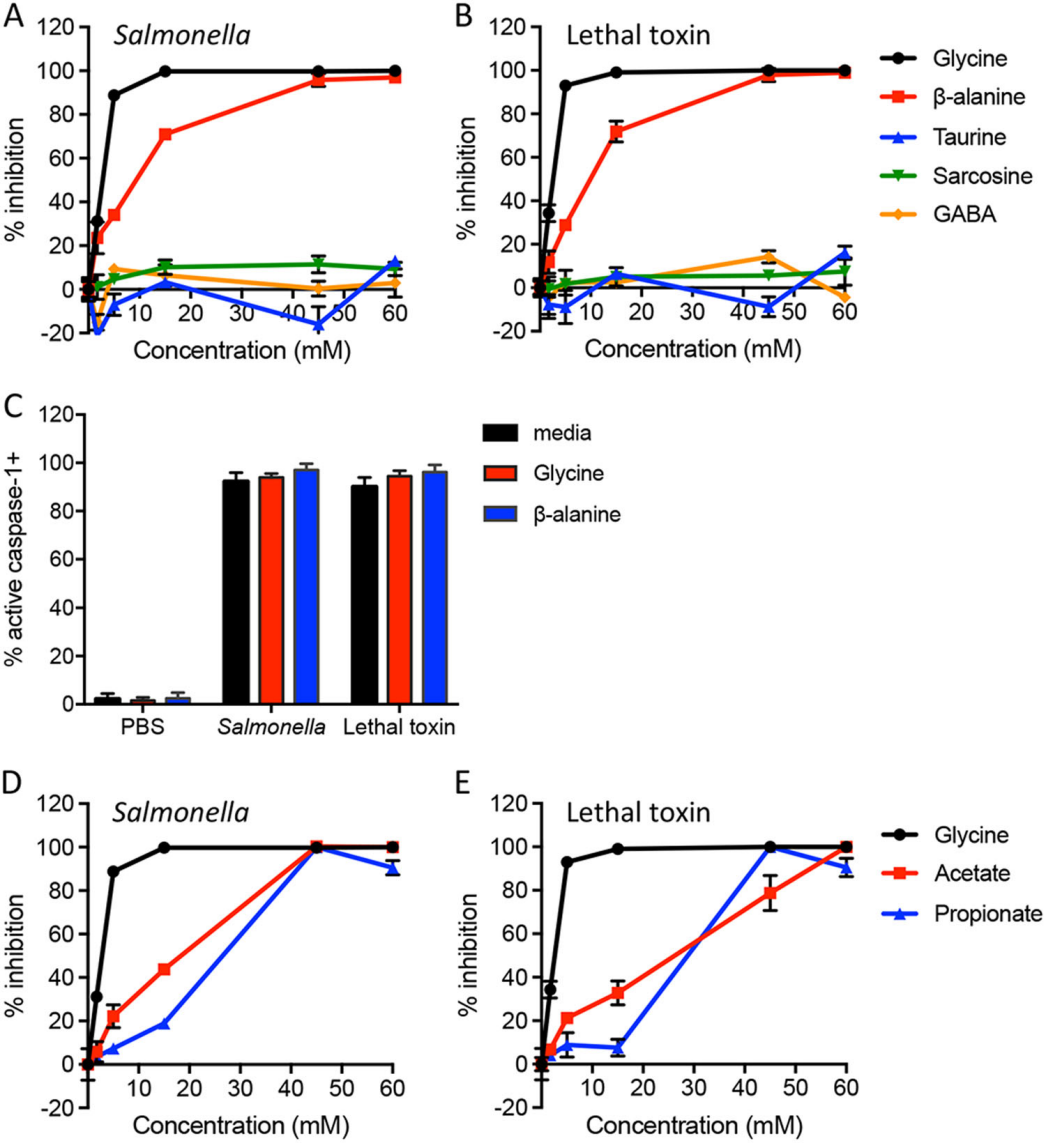
Specific small molecules with structural similarity to glycine inhibit pyroptotic lysis. Bone marrow-derived macrophages were treated with *Salmonella* (**a**, **d**) or anthrax lethal toxin (**b**, **e**) in the presence of glycine or related small molecules that vary at the amino position (titrated from 1.7–60 mM). LDH released during pyroptotic lysis was measured and compared to LDH released in the absence of inhibitor (% inhibition). Representative data (mean ± SD, *n* = 3) from three or more independent experiments are shown. **c** To determine whether glycine or β-alanine block caspase-1 activation, macrophages were treated with PBS, *Salmonella* or anthrax lethal toxin in the presence of medium alone, 5 mM glycine or 15 mM β-alanine. Active caspase-1 was identified by FAM-YVAD-FMK staining. Cumulative data from two independent experiments (mean ± SD, *n* = 7 high power fields with 328–574 total cells queried per condition) are shown

Glycine is a simple amino acid with a single carbon attached to an amino and a carboxyl group. To understand the structural requirements for cytoprotection during pyroptosis, we systematically tested a panel of amino acids and related small molecules for their ability to prevent pyroptotic lysis (Supplemental Fig. S1). We used *Salmonella* infection and anthrax lethal toxin to trigger pyroptosis in murine bone marrow-derived macrophages (BMDMs) via the NLRC4 and NLRP1b inflammasomes, respectively^22^. Pyroptotic lysis was assessed by measuring release of the large cytoplasmic enzyme, LDH. Consistent with prior studies^14^, we observed that glycine prevented LDH release from *Salmonella*-infected and lethal toxin-treated macrophages (Fig. 1a, b and Fig. S2A), without affecting the detection of LDH released from detergent-treated cells (Fig. S3A). We observed robust inhibition with 5 mM glycine (Fig. S2A) and an IC50 of 1.6–1.8 mM (Table S1). Caspase-1 activation, as assessed by labeling with the caspase-1 activity probe FAM-YVAD-FMK^23^, was not affected by glycine (Fig. 1c and Fig. S2B).

We then examined amino group substitutions, and found that β-alanine also inhibited pyroptotic lysis (Fig. 1a, b), albeit with a reduced potency compared to glycine, reaching maximal inhibition at 45 mM (IC50 of 9.5–10.1 mM, Table S1). Similarly to glycine, β-alanine did not prevent caspase-1 activation (Fig. 1c and Fig. S2B), or LDH release from detergent-treated cells (Fig. S3A). However, neither γ-aminobutyric acid (GABA), sarcosine, nor taurine protected against pyroptotic lysis (Fig. 1a, b). These results suggest that there may be molecular size constraints for cytoprotection, as insertion of one methylene group between the carboxyl and amino termini is tolerated (β-alanine), while two are not (GABA). Substitution of the amino group with a simple hydrogen (acetate) or methyl group (propionate) afforded protection against pyroptotic lysis only at higher concentrations (Fig. 1d, e), which did not affect detection of LDH released from detergent-treated cells (Fig. S3B), suggesting that the amino group contributes to protection, but may not be absolutely required.

**Fig. 2:**
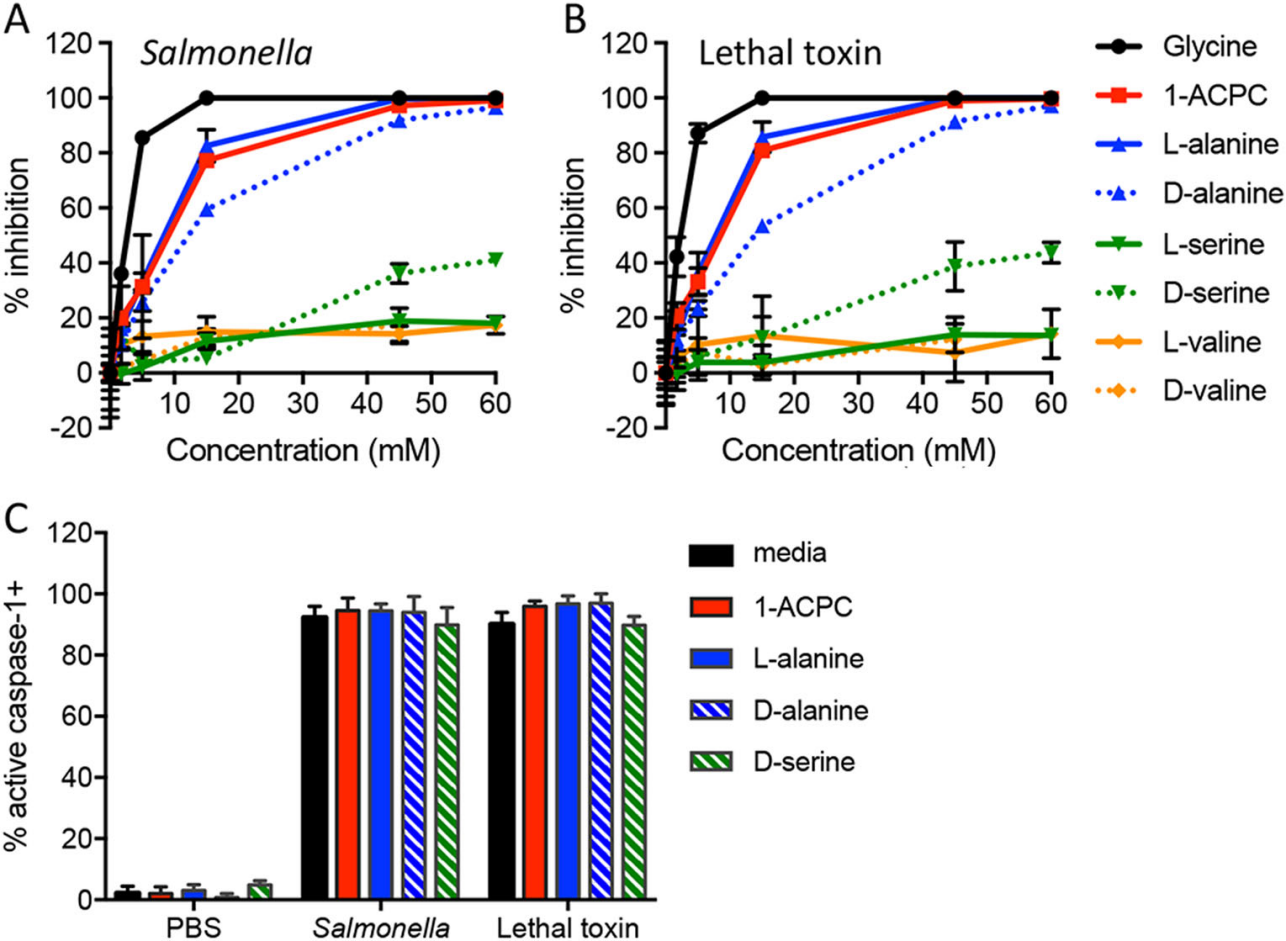
Substitution of amino acid side chains influences cytoprotection. Bone marrow-derived macrophages were treated with *Salmonella* (**a**) or anthrax lethal toxin (**b**) in the presence of glycine or related amino acids with R group substitutions (titrated from 1.7–60 mM). LDH released during pyroptotic lysis was measured and compared to LDH released in the absence of inhibitor (% inhibition). Representative data (mean ± SD, *n* = 3) from three or more independent experiments are shown. **c** Cytoprotective amino acids do not block caspase-1 activation. Macrophages were treated with PBS, *Salmonella* or anthrax lethal toxin in the presence of medium alone, glycine (5 mM), 1-aminocyclopropane carboxylic acid (1-ACPC, 15 mM), l-alanine (15 mM), d-alanine (45 mM), or d-serine (15 mM). Active caspase-1 was identified by FAM-YVAD-FMK staining. Cumulative data from two independent experiments (mean ± SD, *n* = 7 high power fields with 357–588 total cells queried per condition) are shown

We then examined other amino acids with R group substitutions, and found inhibition of pyroptotic lysis by both alanine enantiomers, as well as the cyclic amino acid, 1-aminocyclopropane carboxylic acid (1-ACPC) (Fig. 2a, b and Table S1), without an effect on caspase-1 activation (Fig. 2c) or detergent-mediated LDH release (Fig. S3C). d-serine demonstrated modest protection at higher concentrations, while l-serine and l- and d-valine were without protective effect (Fig. 2a, b). These results suggest that specific substitutions of the hydrogen R group maintain cytoprotective effects, but with lower potency compared to glycine.

**Fig. 3:**
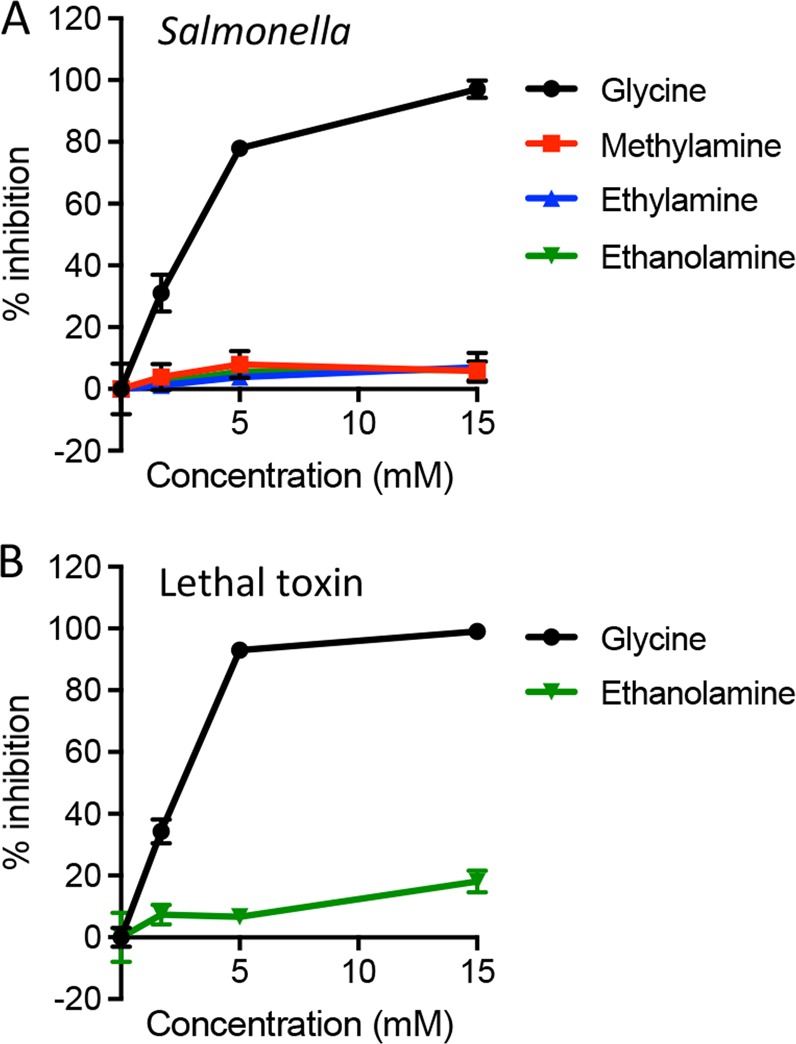
The carboxyl group is essential for protecting cells from pyroptotic lysis. **a** Bone marrow-derived macrophages were infected with *Salmonella* in the presence of glycine, methylamine, ethylamine, or ethanolamine (titrated from 1.7–15 mM). **b** Macrophages were treated with anthrax lethal toxin in the presence of glycine or ethanolamine. LDH released during pyroptotic lysis was measured and compared to LDH released in the absence of inhibitor (% inhibition). Representative data (mean ± SD, *n* = 3) from three or more independent experiments are shown

Finally, we examined carboxyl substitutions, and found no inhibition of *Salmonella*-induced pyroptotic lysis with methylamine, ethylamine, or ethanolamine (Fig. 3a). Methylamine and ethylamine prevent endosome acidification, which is required for anthrax lethal toxin entry^24^ and subsequent induction of pyroptosis (Fig. S4). However, ethanolamine was not protective against lethal toxin-induced pyroptotic lysis (Fig. 3b). These results indicate that the carboxyl group is absolutely required for cytoprotection.

Together, these results reveal a subset of molecules, structurally related to glycine, that provide protection against pyroptotic lysis, with a decreasing potency order of: glycine > β-alanine, 1-ACPC, l-alanine > d-alanine. These findings define specific structural requirements for small molecule-mediated protection against pyroptotic lysis.

### GlyR modulators prevent pyroptotic lysis, but glycine cytoprotection is independent of GlyR

Glycine is well known to bind the glycine receptor (GlyR) and act as an inhibitory neurotransmitter^25^. Ligand binding triggers opening of the GlyR chloride channel, allowing chloride influx to hyperpolarize the neuronal membrane and limit depolarizing signals. Glycine is the classical agonist for GlyR, but other amino acids also have activity with a potency order of: glycine > β-alanine > l-alanine > d-alanine, while GABA fails to activate GlyR^26,27^. This pattern of activity is remarkably similar to our observations for protection against pyroptotic lysis, suggesting the hypothesis that glycine cytoprotection is mediated by GlyR.

GlyR is a pentameric receptor composed of alpha and beta subunits; four differentially expressed alpha subunits and one beta subunit have been identified^28^. The beta subunit interacts with the scaffolding protein gephyrin, and heteropentameric receptors are thought to consist of either two alpha and three beta subunits or three alpha and two beta subunits^29–31^. In heterologous expression systems, alpha subunits alone are sufficient to produce functional homomeric glycine-gated channels^28^. Alpha subunit expression is anatomically and developmentally regulated in the nervous system. Expression of GlyR subunits in nonneuronal cell types has been described, and functional evidence suggests the possibility of GlyR activity in macrophages^32,33^.

**Fig. 4:**
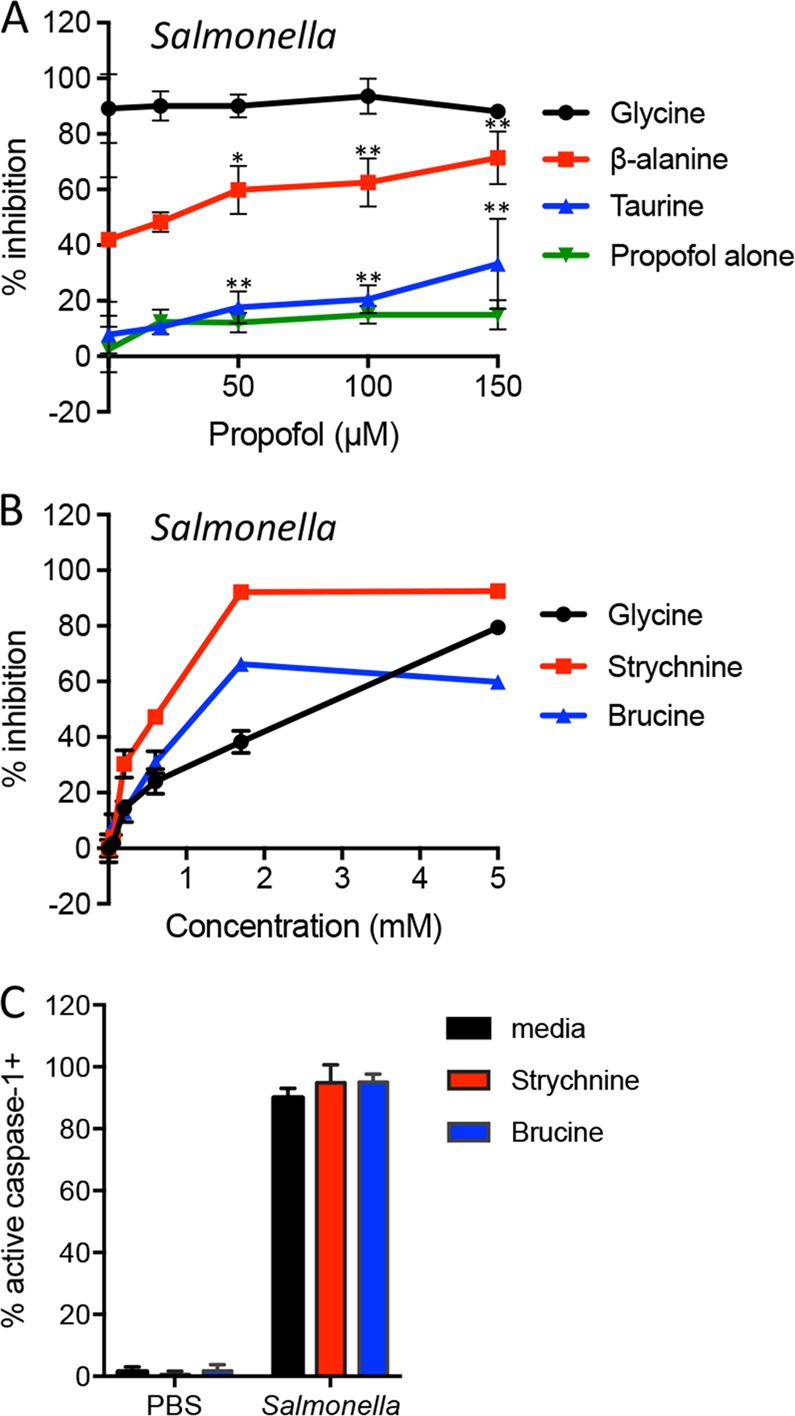
Glycine receptor agonists and antagonists both inhibit pyroptotic lysis. **a** Macrophages were pretreated with increasing concentrations of propofol for 30 min prior to addition of the GlyR agonists, glycine, β-alanine, or taurine (all 5 mM). *Salmonella*-induced LDH release was measured and compared to LDH released by pyroptotic cells in the absence of all inhibitors (% inhibition). Representative data (mean ± SD, *n* = 3) from three or more independent experiments are shown. *P < 0.05, **P < 0.01 (unpaired *t-*test) indicates significance compared to 0 mM propofol. **b** Macrophages were infected with *Salmonella* in the presence of GlyR antagonists, strychnine, or brucine (titrated from 0.06 to 5 mM). Representative data (mean ± SD, *n* = 3) from three or more independent experiments are shown. **c** GlyR antagonists do not block caspase-1 activation. Macrophages were treated with PBS or *Salmonella* in the presence of medium alone, 1.7 mM strychnine, or 1.7 mM brucine. Active caspase-1 was identified by FAM-YVAD-FMK staining. Cumulative data from two independent experiments (mean ± SD, *n* = 7 high power fields with 413–578 total cells queried per condition) are shown

Propofol is an anesthetic that potentiates the GlyR and 500 µM propofol allosterically enhances GlyR responses to the partial agonists, β-alanine, and taurine^34^. We therefore tested the hypothesis that propofol might potentiate the cytoprotective ability of β-alanine and taurine against pyroptotic lysis. Propofol alone afforded no protection against pyroptotic lysis, but enhanced the cytoprotective ability of β-alanine (Fig. 4a). Additionally, taurine alone had no cytoprotective effect (Fig. 1a), but in combination with 150 µM propofol, we observed partial inhibition of pyroptotic lysis (Fig. 4a). Together, these results suggest that protection from pyroptotic lysis mirrors the agonist properties of ligands at the GlyR.

Strychnine (Fig. S5) is the classical GlyR antagonist and blocks glycine-mediated effects at this receptor at nanomolar concentrations^35^. We did not observe antagonism of glycine cytoprotection by strychnine (Fig. S6A), but surprisingly found that strychnine alone mimicked glycine cytoprotection (Fig. S6A and Fig. 4b). Strychnine prevented pyroptotic lysis with increased potency compared to glycine, with an IC50 of 0.5–0.8 mM (Table S1), without affecting caspase-1 activation (Fig. 4c) or detection of LDH released from detergent-treated cells (Fig. S6B). Although strychnine is classically a GlyR antagonist with affinity in the 5–15 nM range^35^, at higher concentrations strychnine can act as an agonist for glycine-gated chloride channels^36,37^. This could potentially explain our observation that strychnine alone provided cytoprotection and synergistically potentiated cytoprotection in presence of glycine. We then examined brucine (Fig. S5), another well-characterized GlyR antagonist^38^. We found that brucine mimicked glycine and strychnine in preventing lysis during pyroptosis without affecting caspase-1 activation or detection of LDH released from detergent-treated cells (Fig. 4b, c and Fig. S6B). The IC50 of brucine (0.6–0.7 mM) was similar to strychnine. Notably, these are much higher than the nanomolar concentrations needed for inhibition at the GlyR.

**Fig. 5:**
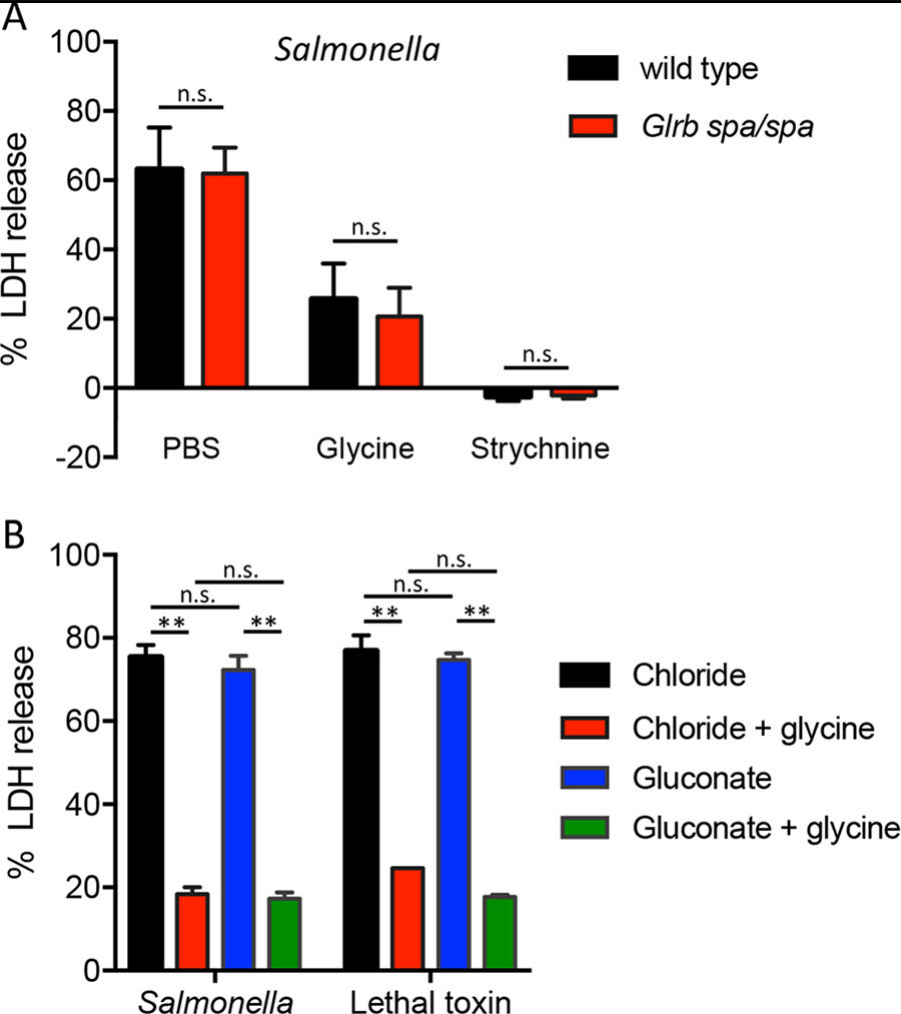
GlyR is not required for glycine-mediated inhibition of pyroptotic lysis. **a** Bone marrow-derived macrophages from wild type and GlyR β subunit-deficient (*Glrb spa*/*spa*) mice were infected with *Salmonella* in the presence or absence of 5 mM glycine or 1.7 mM strychnine and LDH release was measured after 60 min. **b** Wild-type macrophages were treated with *Salmonella* or anthrax lethal toxin in buffer containing either chloride or gluconate anions and LDH release was measured. Representative data (mean ± SD, *n* = 3) from three or more independent experiments are shown. *P < 0.05, **P < 0.01, n.s. nonsignificant (unpaired *t-*test)

These results were consistent with the hypothesis that the GlyR is involved in protection against pyroptotic lysis. To genetically determine whether the GlyR is required for the protective effect of glycine, we cultured macrophages from homozygous *spastic* (*spa*) mutant mice. The *spa* mutation is a 7 kilobase insertion in intron 6 of *Glrb*, causing aberrant splicing and reducing expression of the GlyR beta subunit to undetectable levels^27,39^. Macrophages from *spa*/*spa* mice underwent pyroptotic lysis that was inhibited by both glycine and strychnine (Fig. 5a), suggesting that canonical heteromeric GlyRs containing the beta subunit are dispensable for cytoprotection.

The neuronal GlyR is a heteropentamer comprised of alpha and beta subunits, but in heterologous expression systems, alpha subunits alone are sufficient to produce functional homomeric glycine-gated channels^28^. Although *spa* mutant macrophages lacking the beta subunit exhibit glycine cytoprotection, this does not exclude the possibility of homomeric alpha subunit GlyR channels mediating protection against pyroptotic lysis. Both homo- and hetero-oligomeric GlyR channels share strong selectivity for permeating chloride ions and do not conduct larger gluconate ions^40^. To determine whether glycine protection against pyroptotic lysis requires extracellular chloride conductance, we treated macrophages in medium in which chloride was replaced with larger gluconate anions. We found that macrophages underwent pyroptotic lysis that was blocked by glycine when cultured in either chloride or gluconate-containing medium (Fig. 5b). These findings suggest that glycine protection against pyroptotic lysis does not require extracellular chloride conductance, arguing against an effect mediated by either homo- or hetero-oligomeric GlyR channels. Together, these data refute the implied correlation between observed potency order (glycine > β-alanine > l-alanine > d-alanine) and GlyR function and suggest that these small molecules act at another target to prevent pyroptotic lysis.

### Identification of novel inhibitors of pyroptotic lysis

Strychnine and brucine are specific for GlyR when used at nanomolar concentrations^35^, but protection from pyroptotic lysis requires micromolar concentrations, potentially consistent with lower affinity binding to a related receptor. To identify novel inhibitors of pyroptotic lysis, we performed a small-scale, hypothesis-driven screen of small molecules with known activities at the GlyR and related ligand-gated ion channels (Fig. S5 and Table 1).

**Fig. 6:**
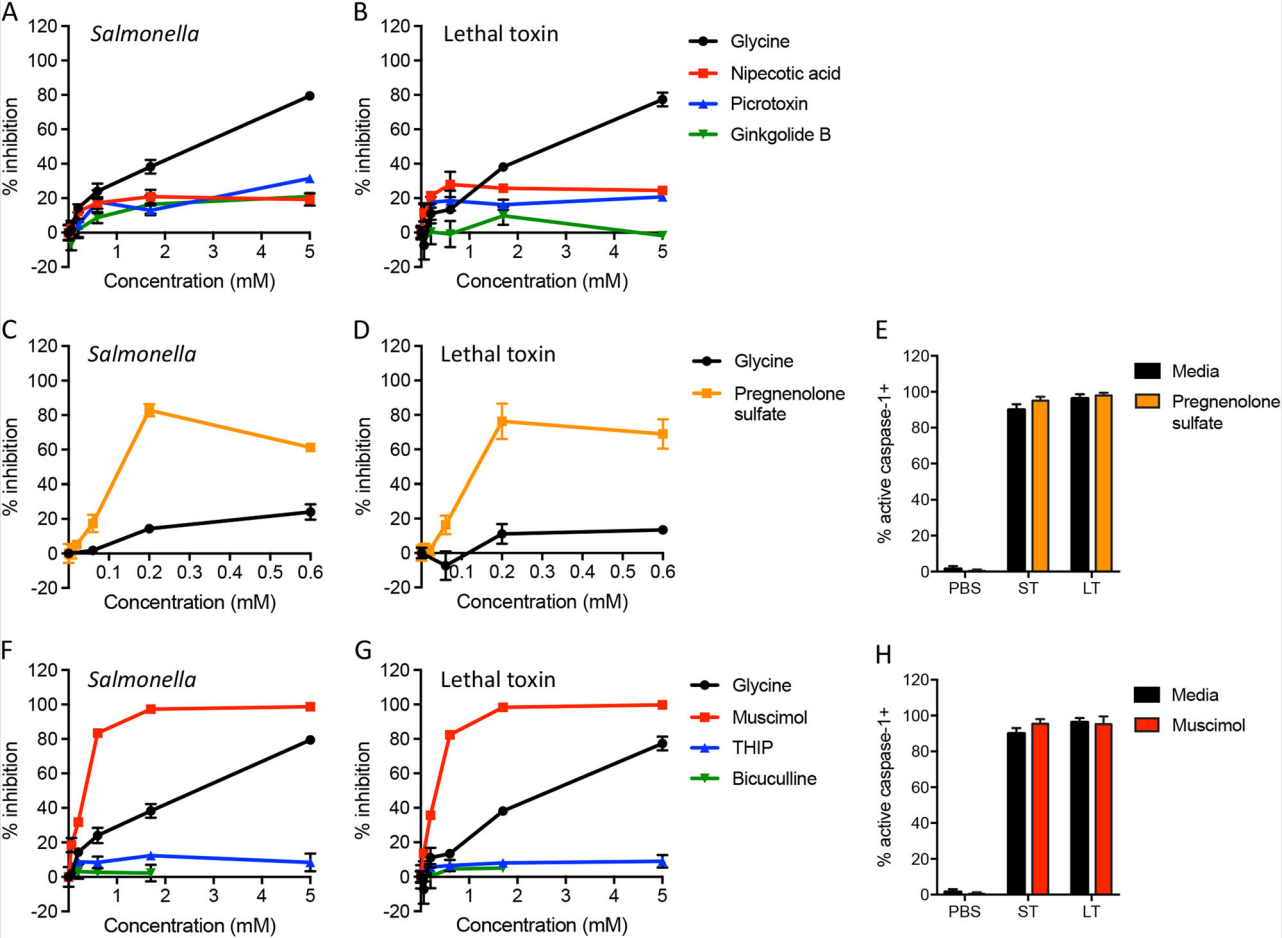
Diverse ligand-gated ion channel modulators prevent pyroptotic lysis. Bone marrow-derived macrophages were treated with *Salmonella* (**a**, **c**, **f**) or anthrax lethal toxin (**b**, **d**, **g**) in the presence of various molecules known to bind GlyR and/or GABA_A_ receptor (titrated from 0.06 to 5 mM; 0.007 to 0.6 mM pregnenolone sulfate). LDH released during pyroptotic lysis was measured and compared to LDH released in the absence of inhibitor (% inhibition). Representative data (mean ± SD, *n* = 3) from three or more independent experiments are shown. **e**, **h** Macrophages were treated with PBS, *Salmonella* (ST), or anthrax lethal toxin (LT) in the presence of medium alone, 0.2 mM pregnenolone sulfate (**e**) or 1.7 mM muscimol (**h**). Active caspase-1 was identified by FAM-YVAD-FMK staining. Cumulative data from two independent experiments (mean ± SD, *n* = 7 high power fields with 421–578 total cells queried per condition) are shown

As we observed inhibition of pyroptotic lysis by the GlyR antagonists strychnine and brucine, we first sought to determine whether other GlyR antagonists prevent pyroptotic lysis. Nipecotic acid is a competitive GlyR antagonist like strychnine and brucine^26^. However, unlike strychnine and brucine, nipecotic acid did not protect against pyroptotic lysis (Fig. 6a, b). In addition, nipecotic acid failed to antagonize the protective effect of glycine and strychnine (Fig. S7). These results suggest that some, but not all, GlyR modulators prevent pyroptotic lysis, consistent with our findings that protection does not require the hetero-oligomeric GlyR channel.

Pregnenolone sulfate is a neurosteroid that inhibits GlyR^41^, but also blocks GABA_A_ receptors^42^. Pregnenolone sulfate potently inhibited pyroptotic lysis induced by both *Salmonella* and lethal toxin (Fig. 6c, d), with an IC50 of 0.06–0.07 mM (Table S1). Pregnenolone sulfate did not affect caspase-1 activation (Fig. 6e) or detection of LDH from detergent-treated cells (Fig. S8B). We then examined other inhibitors of both GlyR and GABA_A_ receptors. We found no significant protection against pyroptotic lysis with the prototypic GABA_A_ receptor antagonist, picrotoxin, which also blocks homomeric GlyR receptors^35^ (Fig. 6a, b). Ginkgolide B also inhibits both GlyR^43^ and GABA_A_ receptors^44^, but was not significantly protective against pyroptotic lysis (Fig. 6a, b).

Given that pregnenolone sulfate acts at GABA_A_ receptors, we then examined additional GABA_A_ receptor modulators. We found no protection against pyroptotic lysis with bicuculline (Fig. 6f, g), a selective GABA_A_ receptor antagonist^35^. However, the classic GABA_A_ receptor agonist, muscimol, potently inhibited pyroptotic lysis induced by both *Salmonella* and anthrax lethal toxin, with an IC50 of 0.3 mM (Fig. 6f, g and Table S1). Muscimol did not affect inflammasome-induced caspase-1 activation or detection of LDH from detergent-treated cells (Fig. 6h and Fig. S8C). In contrast, the conformationally restrained muscimol analog, 4,5,6,7-tetrahydroisoxazolo(5,4-c)pyridin-3-ol (THIP)^45^, did not significantly protect against pyroptotic lysis (Fig. 6f, g). As the protective effect of muscimol is not recapitulated by another GABA_A_ agonist, this curious inconsistency suggests that the GABA_A_ receptor may not mediate muscimol protection. Together, these results reveal a novel panel of diverse small molecules with unexpected potent activity to protect against pyroptotic lysis.

## Discussion

Glycine protection from pyroptotic lysis has long been recognized, but this study is the first to examine the molecular requirements for this effect. Osmoprotectants, large inert molecules that cannot pass through plasma membrane pores, block pyroptotic lysis by balancing osmotic differences between the cytosol and the extracellular milieu. Osmoprotectants were initially used to predict the existence of a caspase-1-dependent plasma membrane pore, prior to the identification of gasdermin D^9^. Glycine has been referred to as an osmoprotectant, however its small size would allow free passage through the large gasdermin D pore, preventing glycine from being able to balance the osmotic forces. Indeed, other similarly sized molecules, such as taurine, serine and ethanolamine do not protect against pyroptotic lysis (Figs. 1–3). Together, our data reveal specific structural requirements for protection, which may be mediated by an unidentified receptor. Alternatively, glycine and structurally similar small molecules may exert a cytoprotective effect by other mechanisms, such as modulating the regulatory volume decrease, which would be expected to engage during pyroptotic cell swelling^8^.

Glycine cytoprotection has been well documented in diverse cell types in other models of cell death, including ATP depletion and oxidant injury (reviewed in ref. ^17^). In these models, glycine protection is reversible and there is no role for glycine metabolism, ATP preservation, intracellular pH regulation, or cytoskeletal stabilization. During pyroptosis, glycine protection is also rapidly reversible^15^ and cellular ATP is not preserved^16^. The profile of molecules that protect against ATP depletion and oxidant injury does not match known GlyRs, but does share some similarities with our findings. For example, similarly to pyroptosis, β-alanine is protective against hypoxic death, while taurine is not^46^. Strychnine also protects against ATP depletion and oxidant injury, and acts at the cell surface^47–49^. More limited data also suggests cytoprotection against hypoxia with pregnenolone sulfate and muscimol^50^. However, in contrast to our findings with pyroptosis, bicuculline also protects against hypoxic cell death^47,48^.

In addition to glycine, we found other amino acids that afford protection against pyroptotic lysis, with a potency order of glycine > β-alanine, l-alanine > d-alanine. This pattern curiously resembles that of known GlyR agonists. However, taurine is an additional well-described GlyR agonist^25^ and afforded no cytoprotection alone (Figs. 1a, b and 4a). Furthermore, d-serine and 1-ACPC protect against pyroptotic lysis but have no agonist activity at GlyR^26,27,51^. Further evidence against a role for GlyR in cytoprotection is the dispensability of the GlyR beta subunit, which is necessary for formation of heteromeric GlyR. Finally, replacement of chloride with gluconate excludes a requirement for extracellular chloride conductance, which is the major function of GlyR^28^. The requirement for the GlyR in glycine protection against hypoxia and oxidant injury has been inconsistent, with some models requiring the GlyR, but most not^17^. Therefore, cytoprotection in different models of cellular injury may not share a common mechanism, but in the case of glycine protection against pyroptotic lysis, we show that GlyR is not required.

In addition to structurally similar small molecules, this study has identified novel cytoprotective compounds with increased potency compared to glycine. These include the canonical GlyR antagonists strychnine and brucine (Fig. 4), the modulatory neurosteroid pregnenolone sulfate, and the canonical GABA_A_ receptor agonist, muscimol (Fig. 6). None of these inhibitors affected caspase-1 activation, indicating that they specifically block pyroptotic lysis downstream of inflammasome activation. All of these are more potent than glycine, but still require concentrations higher than is typically needed for activity at their canonical receptors. The profile of cytoprotection also does not correspond with the classic receptors targeted by these agents, as neither the GlyR inhibitor nipecotic acid nor the GABA_A_ receptor agonists THIP or GABA itself provided protection. Additionally, the GABA_A_ receptor selectively conducts chloride^52^, which is dispensable for protection from pyroptosis, and argues against a role of the GABA_A_ receptor. The GlyR and GABA_A_ receptors are related ligand-gated ion channels, with a number of shared features, including potentiation by propofol^53^. Small molecules with diverse structures act as allosteric modulators of these receptors and alter receptor activity by interacting with binding sites distinct from the primary ligand-binding site^54,55^. We hypothesize that the cytoprotective ability of some GlyR and GABA_A_ modulators may result from modulation of an unidentified receptor with similarity to the GlyR and GABA_A_ receptors. As glycine does not prevent gasdermin D-mediated pore formation^9,16,56^, we hypothesize that glycine and other cytoprotectants do not bind gasdermin D directly, but modulate lysis in conjunction with an additional unidentified protein.

During pyroptosis, glycine prevents rapid membrane disruption, but not IL-1β secretion, pore-mediated small dye uptake, loss of ATP, or induction of secondary cell death pathways; thus, glycine-treated cells do not remain intact indefinitely^9,16,56,57^. Preventing rapid membrane disruption may not ultimately rescue cells from lethality^15^, but may impact the physiologic consequences of cell death by preventing rapid exposure of immunostimulatory intracellular contents, such as actin^58,59^ and mitochondria^60^. Pyroptotic lysis also releases free inflammasomes, which propagate inflammatory responses in bystander cells^61,62^. In addition to promoting inflammation, membrane breakdown may expose “eat-me” signals that promote efferocytosis of pyroptotic cells and clearance of trapped pathogens^63^. Therefore, mechanisms that regulate terminal lysis during pyroptosis may contribute to the inflammatory consequences of this form of cell death, as well as pyroptotic control of infection.

Sepsis is a common and highly fatal condition associated with pyroptosis^2^. In mouse models, septic lethality is mediated by gasdermin D, demonstrating the fatal consequences of rapid pyroptotic lysis^11,64^. Glycine administration is highly protective in models of sepsis^19–21^, suggesting that understanding the mechanism of glycine action may provide novel therapeutic targets for inflammasome-mediated pathology. This study identified novel pyroptotic cytoprotectants of much higher potency than glycine, which may be useful for future studies of pyroptosis.

## Materials and methods

### Cell culture

BMDMs were cultured from wild-type Balb/c mice (Jackson Labs) for 7 days at 37 °C in 5% CO_2_ in DMEM supplemented with 10% FCS, 5 mM HEPES, 0.2 mg/ml l-glutamine, 0.05 mM β-mercaptoethanol, 50 µg/ml gentamicin sulfate, 100 U/ml penicillin and streptomycin, and 30% L-cell-conditioned medium. Macrophages were collected by washing with ice-cold PBS containing 1 mM EDTA and resuspended in supplemented antibiotic-free DMEM (without phenol red) containing 5% FCS (DMEM-5). For the GlyR β subunit experiment, BMDM were cultured from wild-type C57BL/6 and B6.Cg-Glrb^spa^/J mice (JAX #000066) (Jackson Labs). To test the importance of GlyR chloride channel function, macrophages were washed with and incubated in NaCl-HBSS or gluconate-HBSS: 138 mM NaCl (or Na gluconate), 5 mM KCl (or K gluconate), 1.8 mM CaCl_2_ (or Ca gluconate), 4.2 mM NaHCO_3_, 0.34 mM NaH_2_PO_4_-H_2_O, and 0.44 mM KH_2_PO_4_.

### Lactate dehydrogenase (LDH) assay

Macrophages were pretreated for 30 min with glycine or one of the other small-molecule inhibitors at the indicated dose. All chemicals were purchased from Sigma-Aldrich, with the exception of propofol (EMD Millipore). Late-log cultures of *Salmonella typhimurium* SL1344 grown in L-broth containing 0.3 M NaCl were used to infect macrophages (MOI 10:1) for 90 min, unless otherwise indicated. Cells were treated with anthrax lethal toxin comprised of 1 µg/ml protective antigen and 1 µg/ml lethal factor (List Biological) for 2 h^14^. Cells were treated with the detergent-based lysis buffer included in the Cytotox 96 Kit (Promega) for 30 min. Release of cytoplasmic LDH was determined from triplicate samples using the Cytotox 96 Kit and calculated as 100 (experimental LDH − spontaneous LDH)/(maximum LDH −spontaneous LDH).

### Fluorescence microscopy

Macrophages were seeded onto glass coverslips at a density of 2 × 10^5^ cells/well (24-well plate) prior to infection with *S. typhimurium* or treatment with anthrax lethal toxin. Active caspase-1 was detected using a caspase-1-specific probe^23^. FAM-YVAD-FMK (Immunochemistry Technologies) was added to a final concentration of 5 μM for the last 60 min of treatment. Cells were washed twice to remove unbound probe, then fixed and stained with the nuclear dye To-Pro-3 (Molecular Probes). Multiple fields were examined using a Leica SL confocal microscope (W.M. Keck Microscopy Center), and the percent of positive cells was determined using 3–10 fields for each experimental condition.

### Statistics

Data were analyzed by unpaired two-tailed Student’s *t*-test. IC50 values were determined using nonlinear regression and the [Inhibitor] vs. response – variable slope equation in Prism software (GraphPad).

## Supplementary information

Supplemental Material
