# Supplementary material for: Diverse small molecules prevent macrophage lysis during pyroptosis
Source: Cell Death Dis. 2019 Apr 11;10(4):326. doi: 10.1038/s41419-019-1559-4 (PMC6459844; doi:10.1038/s41419-019-1559-4)
Supplement: Supplementary file 1 — Supplemental Material [file 41419_2019_1559_MOESM1_ESM.pdf]

Supplementary Information for:

Diverse Small Molecules Prevent Macrophage Lysis During Pyroptosis

Wendy P. Loomis[1], Andreas B. den Hartigh[1], Brad T. Cookson[1, 2] and Susan L. Fink[1]

[1]Department of Laboratory Medicine, University of Washington, Seattle WA
[2]Department of Microbiology, University of Washington, Seattle WA

Table S1. IC50 for protective molecules (mM)

	Salmonella	Lethal toxin
Glycine	1.8	1.6
β-alanine	9.5	10.1
L-alanine	8.1	7.0
1-ACPC	8.9	8.2
D-alanine	14.0	18.7
D-serine	39.7	43.5
Strychnine	0.5	0.8
Brucine	0.6	0.7
Pregnenolone sulfate	0.07	0.06
Muscimol	0.3	0.3

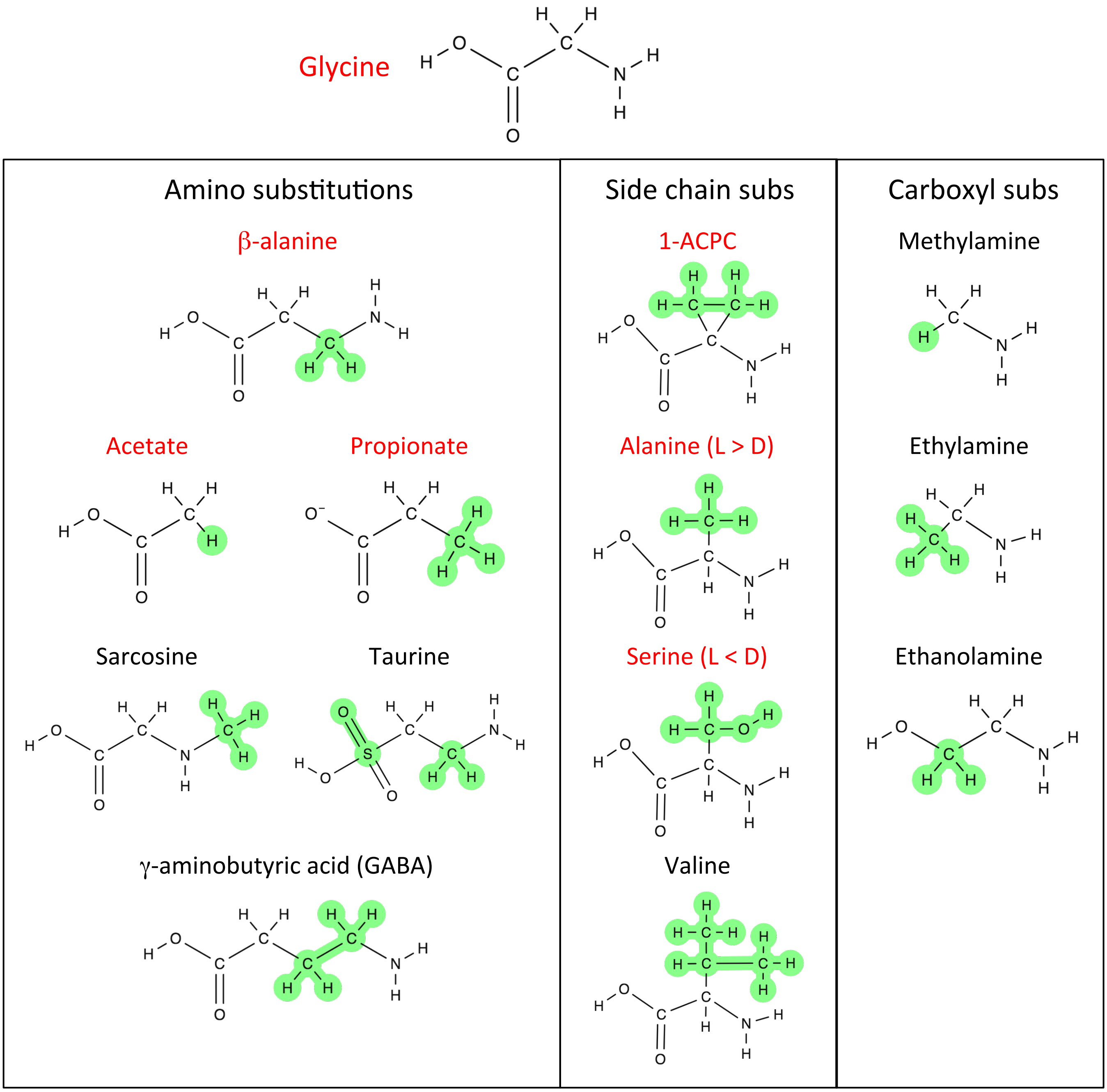

Figure S1. Structural determinants of small molecule inhibitors of pyroptotic lysis. Shown are the molecular structures of amino acids and other related small molecules tested for the ability to prevent pyroptotic lysis of cultured macrophages. Elements of each structure that differ from glycine are highlighted in green. Red names indicate protective compounds.

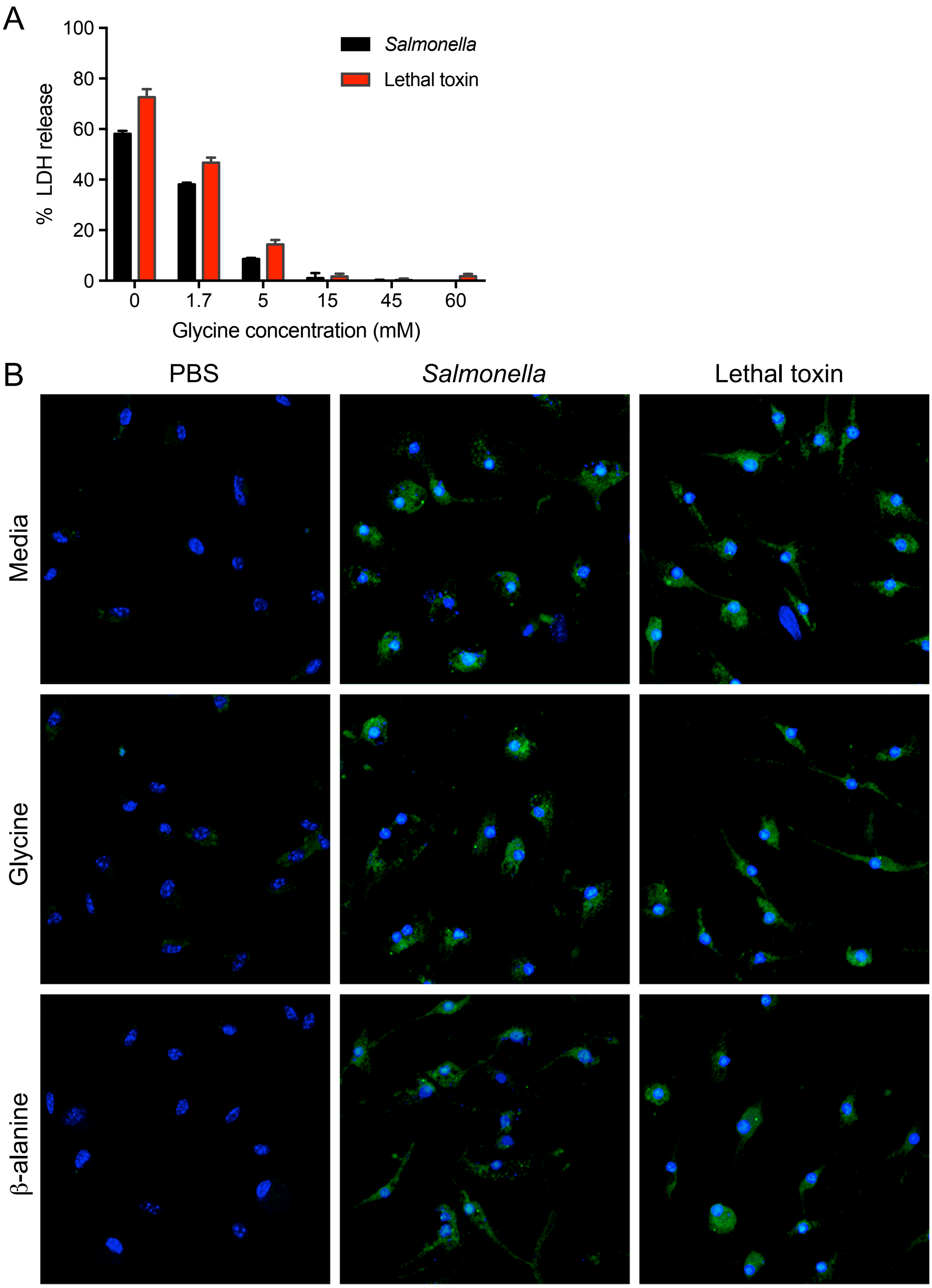

Figure S2. *Salmonella* and anthrax lethal toxin are potent inducers of pyroptosis. (A) Bone marrow-derived macrophages were treated with *Salmonella* (90 min) or anthrax lethal toxin (120 min) in the presence or absence of glycine. Release of cytoplasmic LDH from pyroptotic cells was blocked by glycine in a dose-dependent manner. Representative data (mean +/- SD, n=3) from 3 or more independent experiments are shown. (B) To determine whether glycine or β-alanine block caspase-1 activation, macrophages were treated with PBS, *Salmonella* or anthrax lethal toxin in the presence of medium alone, 5mM glycine or 15mM β-alanine. Active caspase-1 was identified by FAM-YVAD-FMK staining (green). Caspase-1-dependent nuclear condensation was identified using the nuclear dye To-Pro-3 (blue). Representative images from 2 independent experiments are shown.

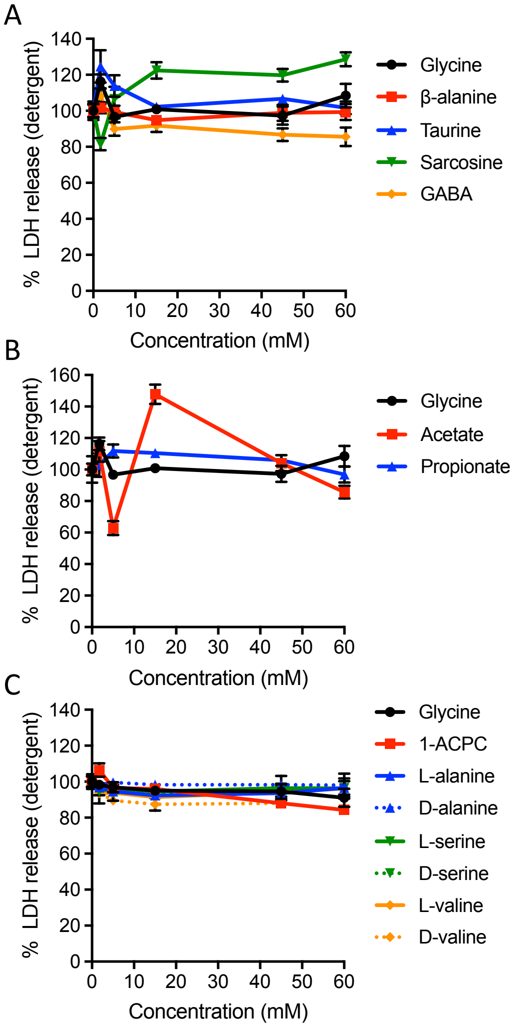

Figure S3. Small molecule inhibitors do not block detergent-mediated cell lysis. Bone marrow-derived macrophages were incubated in the presence of glycine or related small molecules (titrated from 1.7-60mM) as part of the LDH release assays shown in Figs 1, 2. Cells were treated with detergent-based lysis buffer for 30 minutes prior to LDH measurement. LDH release is expressed as a percentage relative to control cells in the absence of any small molecule inhibitors. Representative data (mean +/- SD, n=3) from 3 or more independent experiments are shown.

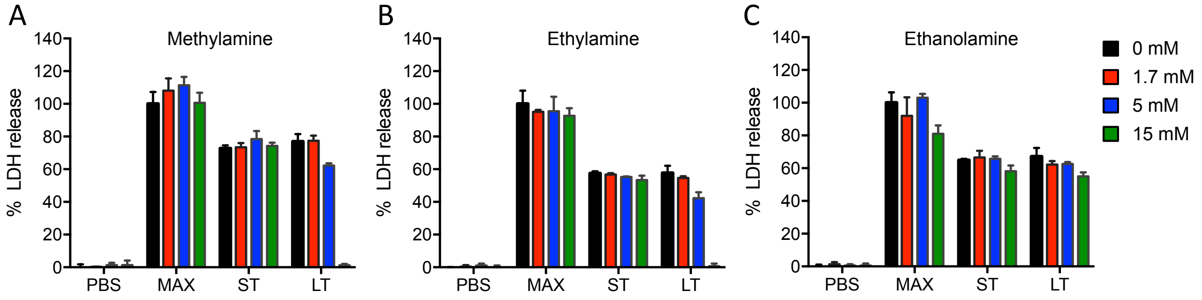

Figure S4. Glycine-related molecules with carboxyl substitutions do not block detergent-mediated cell lysis. Bone marrow-derived macrophages were pre-incubated in media containing (A) methylamine, (B) ethylamine or (C) ethanolamine (titrated from 1.7-15mM). Cells were then treated with either *Salmonella* (ST), anthrax lethal toxin (LT) or detergent-based lysis buffer (MAX) prior to LDH measurement. LDH release is expressed as a percentage relative to control cells in the absence of any small molecule inhibitors. Representative data (mean +/- SD, n=3) from 3 or more independent experiments are shown.

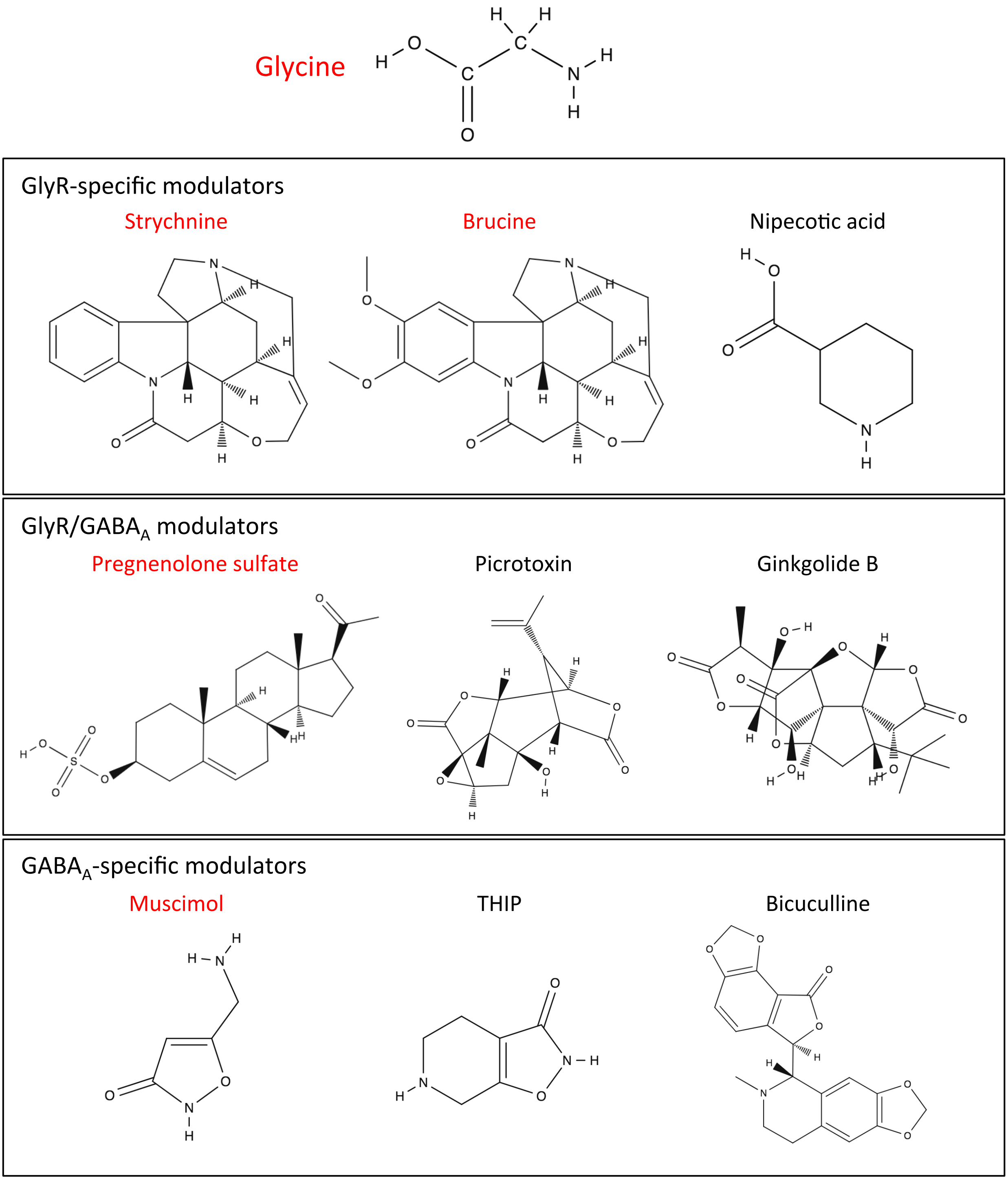

Figure S5. Structure of neuroactive small molecules tested as inhibitors of pyroptotic lysis. Shown are the molecular structures of small molecules with known activities at the GlyR receptor, $GABA_A$ receptor, or both. Each molecule was tested for the ability to prevent pyroptotic lysis of cultured macrophages. Red names indicate protective compounds.

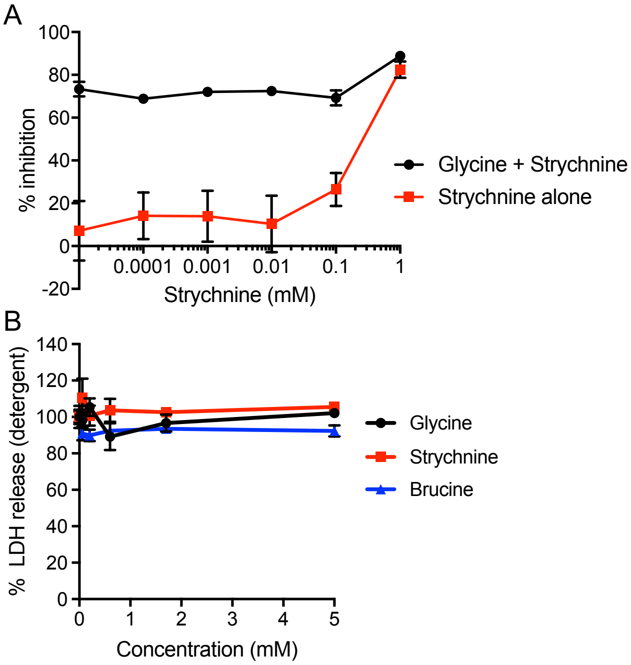

Figure S6. Strychnine does not antagonize glycine-mediated cytoprotection. (A) Bone marrow-derived macrophages were infected with *Salmonella* in the presence of increasing concentrations of strychnine in the presence or absence of glycine (5mM). LDH release was measured and compared to LDH released by pyroptotic cells in the absence of all inhibitors (% inhibition). Representative data (mean +/- SD, n=3) from 3 or more independent experiments are shown. (B) Macrophages were incubated in the presence of glycine, strychnine or brucine (titrated from 0.06-5mM) as part of the LDH release assays shown in Fig 4. Cells were treated with detergent-based lysis buffer for 30 minutes prior to LDH measurement. LDH release is expressed as a percentage relative to control cells in the absence of any small molecule inhibitors. Representative data (mean +/- SD, n=3) from 3 or more independent experiments are shown.

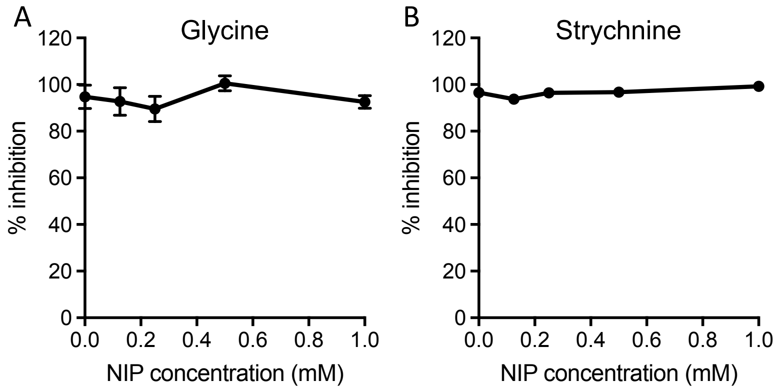

Figure S7. The GlyR antagonist nipecotic acid does not antagonize protection by glycine and strychnine. Macrophages were treated with increasing doses of nipecotic acid for 30 min prior to addition of (A) 5mM glycine or (B) 1.7mM strychnine. *Salmonella*-induced LDH release was measured and compared to LDH released by pyroptotic cells in the absence of all inhibitors (% inhibition). Representative data (mean +/- SD, n=3) from 3 or more independent experiments are shown. All comparisons are non-significant (unpaired *t* test) compared to 0mM nipecotic acid.

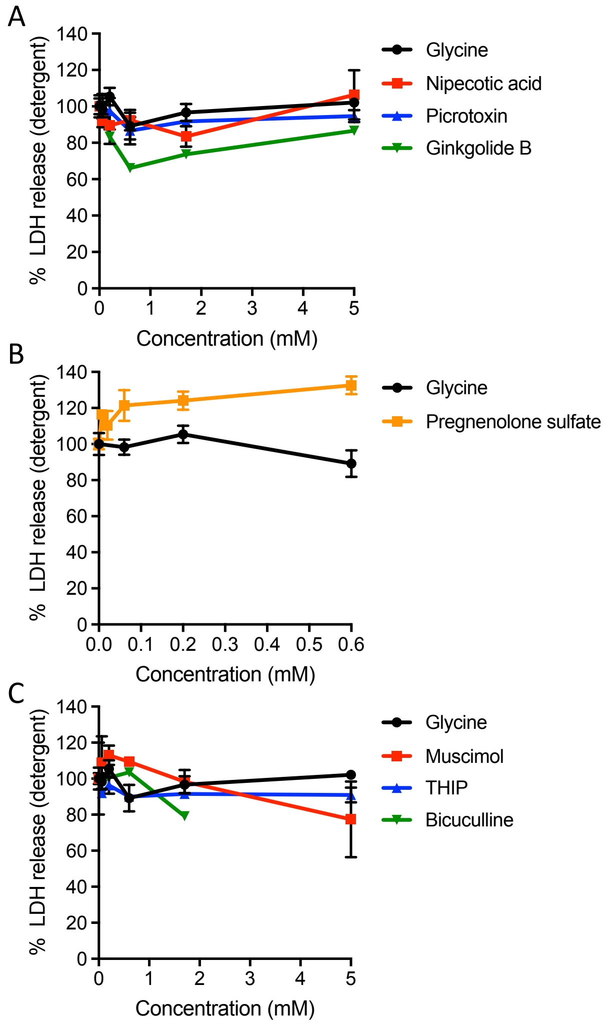

Figure S8. Neuroactive ligands do not block detergent-mediated cell lysis. Bone marrow-derived macrophages were incubated in the presence of various molecules known to bind to GlyR and/or $GABA_A$ receptor (titrated from 0.06-5mM) as part of the LDH release assays shown in Fig 6. Cells were treated with detergent-based lysis buffer for 30 minutes prior to LDH measurement. LDH release is expressed as a percentage relative to control cells in the absence of any small molecule inhibitors. Representative data (mean +/- SD, n=3) from 3 or more independent experiments are shown.
